# Supplementary material for: Functional recovery of the adult murine hippocampus after cryopreservation by vitrification
Source: Proc Natl Acad Sci U S A. 2026 Mar 3;123(10):e2516848123. doi: 10.1073/pnas.2516848123 (PMC12974479; doi:10.1073/pnas.2516848123)
Supplement: Supplementary file 1 — Appendix 01 (PDF) [file pnas.2516848123.sapp.pdf]

**Supporting Information for**

# Functional recovery of the adult murine hippocampus after cryopreservation by vitrification

Alexander German[1]*, Enes Yağız Akdaş[2,3], Cassandra Flügel-Koch[4], Ezgi Erterek[5], Renato Frischknecht[5], Anna Fejtova[2], Jürgen Winkler[1], Christian Alzheimer[6], Fang Zheng[6]

*Corresponding author Alexander German.
Email: alex.german@fau.de

**This PDF file includes:**

- Supporting text
- Figures S1 to S15
- Tables S1 to S7
- Legends for Movies S1 to S12
- SI References

**Other supporting materials for this manuscript include the following:**

- Movies S1 to S12
- Suppl_Movies

## Supporting Information Text

## Materials and Methods

**Animals.** Adult C57BL/6J mice (3-9-month-old) were used in this study, unless otherwise stated. Mice were maintained under standard laboratory conditions, which included a 12/12 light/dark cycle with lights on at 06:00, a temperature of 22 ± 1 °C, and 60% humidity, with water and food available ad libitum. All experiments were conducted in accordance with the guidelines for the Care and Use of Laboratory Animals set by the Local Government of Unterfranken and the European Communities Council Directive (2010/63/EU, RUF-55.2.2-2532-2-2147-13).

**Brain slice preparation, vitrification, and rewarming.** Horizontal slices containing the hippocampus were prepared from mice anesthetized with isoflurane, as described previously (1). Briefly, 350 µm thick brain slices were cut using a Leica VT1200 Vibratome (Leica Biosystems, Germany) in ice-cold sucrose-based artificial cerebrospinal fluid (aCSF) containing (in mM) 75 sucrose, 87 NaCl, 3 KCl, 0.5 $CaCl_2$, 7 $MgCl_2$, 1.25 $NaH_2PO_4$, 25 $NaHCO_3$ and 10 D-glucose. Slices were incubated in the same solution for 10 min at 35 ºC and then maintained in aCSF containing (in mM) 125 NaCl, 3 KCl, 1 $CaCl_2$, 3 $MgCl_2$, 1.25 $NaH_2PO_4$, 25 $NaHCO_3$ and 10 D-glucose at room temperature (RT). All solutions were constantly gassed with 95% $O_2$ - 5% $CO_2$ to keep pH constant (7.4).

After a 30-minute recovery phase, the slices were transferred into a Netwell polyester mesh insert (Corning, USA) for 6-well plates with mesh size 74 µm. The 6-well plate was positioned on the surface of a water bath inside a polystyrene box (set to 10 °C by mixing tap water and ice). Brain slices within the mesh insert were immersed into 10 °C solutions of increasing concentration of cryoprotectant in a carrier solution. During protocol optimization, we successfully used the "Cryonics Institute Vitrification Mixture one" (CI-VM-1) carrier solution (2) and the "Lactose Mannitol five" (LM5) carrier solution (3, 4). CI-VM-1 consists of KCl (28 mM), glucose (230 mM), and organic TRIS-HCl buffer (10 mM) in water. All results reported herein were generated with LM5, which was prepared by dissolving glucose (90 mM), mannitol (45 mM), lactose (45 mM), KCl (8.2 mM), $K_2HPO_4$ (7.2 mM), reduced glutathione (5 mM), adenine (1 mM), $NaHCO_3$ (10 mM) in water (**table S1*)***.

Subsequently, the mesh insert carrying the slices was transferred into higher concentrations of the vitrification solution, which was set to -10 °C in a second polystyrene box containing a 65% w/v ethylene glycol in water bath (kept at -10 °C using dry ice). During protocol optimization, we successfully used a 61% w/v ethylene glycol vitrification solution (5), a 37.5% w/v ethylene glycol and 12.5% dimethyl sulfoxide vitrification solution (2), and a reduced version of the "Vitrification Mixture three" (VM3) vitrification solution detailed below (3). Addition of dimethyl sulfoxide shortened incubation times necessary for vitrification. VM3 contains 22.3% w/v dimethyl sulfoxide, 16.84% w/v ethylene glycol, 12.86% w/v formamide, 7% w/v polyvinylpyrrolidone K12, 1% w/v "Supercool X-1000" ice blocker (6), and 1% w/v "Supercool Z-1000" ice blocker (7). We were successfully able to omit the non-permeating "Supercool" polymers from the composition of our final vitrification solution, which we termed "V3" to refer to VM3 and due to its composition of three permeating solvents. The composition of V3 is also given in (**table S2**). As V3 resulted in the best functional recovery, the other CPA compositions were not explored further. All results reported herein were generated with V3. The w/v concentrations of V3 corresponded to 2.85 M dimethyl sulfoxide, 2.71 M ethylene glycol, 2.86 M formamide, or 8.42 M permeable CPA in total plus 23-35 mM impermeable CPA. 59 g of this solution had a volume of approximately 53.3 ml at 20 °C, and repeated filling and mixing to a total volume of 100 ml required the addition of approximately 49.4 ml of LM5. To assess vitrification stability and toxicity of V3 further, slices were also incubated with final concentrations of 30% w/v = 4.28 M, 45% w/v = 6.42 M, 52% w/v = 7.42 M, 53% w/v = 7.57 M, 54% w/v = 7.71 M, 55% w/v = 7.85 M, 56% w/v = 8.00 M, 57% w/v = 8.14 M, 58% w/v = 8.28 M and 65% w/v = 9.28 M total V3 CPA and permeable V3 CPA, respectively.

**Fig. 1A** illustrates the final vitrification protocol: For CPA loading, brain slices were immersed into 10 ml of 10 °C and -10 °C solutions of increasing concentration of cryoprotectant in the LM5 carrier solution: 0% w/v V3 for 2 min, 2% for 2 min, 4% for 2 min, 8% for 2 min, 16% for 2 min and 30% w/v V3 for 5 min each at 10 °C, followed by 45% w/v V3 for 2 minutes and three iterations of 59% w/v V3 for 5 minutes each at -10 °C.

After vitrification on a copper cylinder (**fig. S1**), the mesh insert was slowly transferred into liquid nitrogen at -196 °C, and subsequently transferred into a -150 °C freezer for storage (up to 7 days). For rewarming, slices were slowly transferred into liquid nitrogen again, and subsequently transferred on top of the copper cylinder again. After 5 minutes, the mesh insert was rapidly rewarmed in 100 ml of 52% w/v V3 at -10 °C.

For CPA unloading, brain slices were immersed into -10 °C and 10 °C solutions of decreasing concentration of V3 in the carrier solution with additional 300 mM mannitol, followed by a final step of carrier solution without added mannitol:
45% w/v V3 for 2 min and 30% at -10 °C for 5 min, followed by 16% w/v V3 for 10 min, 8% for 3 min, 4% for 3 min, 2% for 3 min, 0% for 10 min at 10 °C each, followed by a final step of LM5 without added mannitol at 10 °C for 10 min (**table S3**).

**Cerebral vitrification *in situ*.** The vascular system was cleared from blood via transcardial perfusion of 20 mL of 4°C phosphate-buffered saline (PBS, 1x) injected manually over 2 minutes with a 21G needle and syringe into the left ventricle following incision of the right atrium. PBS composition was NaCl (137 mM), KCl (2.7 mM), $KH_2PO_4$ (1.5 mM), $Na_2HPO_4$ (8.1 mM), pH 7.4. Subsequently, the descending aorta was clamped at the diaphragm with large hemostatic forceps and all caudal tissues removed. The resulting cranial cephalothoracic specimen was transferred to a 4°C PBS bath. The left ventricle was opened via resection of the cardiac apex under a stereomicroscope and a 0.8 mm diameter button cannula (Acufirm Ernst Kratz, Germany) was advanced into the aortic valve in a manner that its equator did not penetrate beyond the atrioventricular plane to allow nondestructive retraction in subsequent steps (**fig. S12A**). The cannula was secured in place with small hemostatic forceps with curved serrated jaws (BH109R, Aesculap, Germany). These steps took 8 minutes to complete.

CPA loading via perfusion was then initiated for 8 minutes at 1 mL/min vitrification solution delivered via a peristaltic pump (Reglo, Ismatec, Germany). The button cannula was then retracted nondestructively, which was followed by a craniectomy on wet ice via resection of the scalp and incision of the skull from the foramen magnum along the sagittal suture with surgical scissors. Damage to the cerebral vasculature and parenchyma could be entirely prevented due to cerebral dehydration during the preceding CPA perfusion (**fig. S13A** vs. **B**). Craniectomy took 4 minutes to complete. Following nondestructive aortic cannulation as described above, the craniectomized cephalothoracic specimen was then subjected to 3 minutes of perfusion with LM5 with added 0.01% w/v methylene blue at a flow rate of 3 mL/min leading to cerebral rehydration (**fig. S13C**). Subsequently, the brain was perfused with 1 ml/min vitrification solution for 25 minutes in a 4°C vitrification solution bath (**fig. S13D**).

All instruments were removed and the specimen was immersed in -20°C 65% w/v V3 vitrification solution for 2 minutes followed by immersion in -140°C isopentane, which was performed by first stirring the cephalon of the specimen before complete immersion. Specimens were kept at -140°C for 1 to 8 days, and were removed from isopentane immersion 2 hours before rewarming. Rewarming was performed by stirring the sample in 20°C 65% w/v V3 vitrification solution for 1 minute followed by immersion in 4°C 59% w/v V3 vitrification solution for 5 minutes.

The specimen was subsequently transferred into a 4°C washout solution bath (10% w/v dextran 70 in LM5) and the aorta was cannulated irreversibly by penetrating the atrioventricular plane with the equator of the button cannula (**fig. S12B**). The large forceps at the diaphragm to close the descending aorta was connected to the small cardiac forceps to secure the button cannula in the ascending aorta with two 30x3mm neodymium magnet disks to enable rotation of the specimen in

subsequent steps for cerebral inspection. Perfusion was initiated with the washout solution (10% w/v dextran 70 in LM5) at 0.3 mL/min for 10 minutes (**fig. S13E**), followed by 0.4 mL/min for 5 minutes and 0.5 mL/min for 10 minutes. The perfusion was continued until the cerebrum was judged to have attained normal size, which took another 5 to 10 minutes (**fig. S13F**).

After completion of perfusion, the brain was extracted and immersed into 4°C LM5 and subjected to vibratome slicing as described above, including an intermediate step in aCSF at RT for 1 hour to improve recovery. 0.01% w/v methylene blue was added to the LM5 interleaved loading and initial washout solution for improved process control (**fig. S12C**). An overview of the protocol is documented in **table S4** and visualized **fig. S14**.

The surgical protocol was prone to failure primarily due to aortic rupture or insufficiency of the aortic valve with reflux of the washout solution into the pulmonary circulation. In a subset of cases, the brain did not undergo a volume reduction after the second CPA perfusion and regained its normal volume instead, with no visual gap between the cerebral hemisphere and the skull base (**Fig. 4Aiii**). These specimens were excluded from further processing as this was a predictor for cerebral edema after CPA washout, as in **Fig. 4Aiv**. Representative images of these failure cases are shown in **fig. S12D-G**, and counts of failures in **table S5**.

During protocol development, we explored BBB opening via perfusion of the detergent sodium dodecyl sulfate (SDS, (2)), perfused for 30 seconds at 4°C at a concentration of 100mg/l, which resulted in the formation of extensive peripheral and cerebral edema, and 10mg/l, which resulted in extensive peripheral edema combined with unmitigated cerebral dehydration. Trials to sonicate the whole specimen during perfusion with SonoVue® microbubbles (8, 9) resulted in extensive peripheral edema and was not pursued further. BBB opening attempts with 20% w/v mannitol (10), proteases trypsin and papain perfused for 10 minutes at RT, calcium chelators EDTA and EGTA perfused for 2 hours at RT (11), and high-pressure perfusion (12) did not mitigate cerebral dehydration in our hands. We explored the approach of increasing intracerebral osmolarity upfront via the low-affinity, high-capacity brain glucose transport (13) by perfusing with 1M glucose for 10 minutes at RT. We attempted modification of BBB osmoregulation (14) via the controversial aquaporin 4 inhibitors (14-17) acetazolamide (18), tetraethylammonium, AqB013, TC AQP1 and TGN-020 (17) at ten times their reported IC50 concentration, perfused for 10 minutes at RT before CPA shock-loading with the vitrification solution with the respective compound added. These compounds did not mitigate cerebral dehydration in our hands. Given the reported effects of TGN-020, it may be beneficial to administer this compound *in vivo*, e.g., 20 min before sacrifice and CPA shock-loading (17, 19). We also perfused with vitrification solution during 30-second pulses alternating with 90-second pauses for 40 minutes to mitigate differential astrocyte dehydration, but did not observe recovery. Incidentally, we observed that after storage for 12 hours at 4°C, no cerebral volume reduction occurred during CPA perfusion. When using 20% w/v PVP K12 instead of dextran for hyperoncotic washout, this resulted in extensive brain slice swelling post-slicing. Prolonged perfusion with PBS after hyperoncotic washout resulted in cerebral edema. Already by the end of the initial 8-minute CPA perfusion step (**fig. S14B**), the brain appeared to undergo vitrification when submerged and subsequently deliberately fractured to pieces in liquid nitrogen. Some fPSP response could be elicited after CPA loading via perfusion, slicing in 30% V3 in LM5 with added mannitol, and resumption with the gradual slice unloading protocol as described above and in **table S3**.

**Measurement of temperature and estimation of tissue-level cooling and rewarming rates**

During the slice vitrification protocol, sample temperature was continuously recorded throughout gradual CPA loading and washout (**fig. S6A**). Target temperatures of 10 °C during loading and −10 °C during washout were maintained with a deviation restricted to ±1 °C. This was achieved by manual addition of blue ice to maintain the 10 °C bath, and dry ice to the ethylene glycol/water bath to maintain −10 °C.

Apparent vitrification of slices was visually indicated after <2 seconds after placement on the copper cylinder via a plasticized surface. Apparent liquefaction was equally determined to occur <2 seconds after immersion in -10°C V3 solution.

To determine a lower limit for the cooling rate achievable during cerebral vitrification, a 0.5 ml Eppendorf tube was filled with 327 mg of V3 solution to simulate the brain exposed by craniectomy after interleaved CPA equilibration. A thermocouple was positioned at the geometric center of the solution. This phantom was equilibrated in a 4 °C bath and then rapidly transferred into a −140 °C isopentane bath. The temperature trajectory allowed quantification of the time spent in the metastable region defined by the melting temperature ($T_m$ = −40.8 °C) and the glass transition temperature ($T_g$ = −128.3 °C) (20). The phantom traversed this interval in 35.7 s, corresponding to an average internal cooling rate of 2.45 °C/s at the center of the phantom (**fig. S6B**).

A lower limit for the rewarming rate was determined using the same phantom. After vitrification in the −140 °C isopentane bath, the tube was transferred into a 4 °C bath containing V3 solution. The time spent between $T_g$ and $T_m$ during rewarming was 21.3 s, corresponding to an average rewarming rate of 4.11 °C/s at the center of the phantom (**fig. S6C**).

To provide heat transfer estimates, we assumed a thermal conductivity $k \approx 0.5\ W \cdot m^{-1} \cdot K^{-1}$ and a thermal diffusivity $\alpha \approx 1 \times 10^{-7}\ m^2/s$. For the slice preparation, cooling and rewarming are dominated by conduction from below through a nylon mesh saturated with CPA (assumed thickness 100 µm) and into the acute slice (thickness 350 µm). The bottom of the mesh (x = 0) was treated as a fixed-temperature boundary representing the copper block, while the top of the slice ($x = d_{tot}$) was treated as approximately adiabatic ($\partial T/\partial x = 0$). Using an explicit finite-difference solution of the transient heat equation under these boundary conditions, we calculated the temperature history at the top of the slice for a cooling step from −10 °C (uniform initial condition) to a −196 °C block, resulting in a residence time of 0.69 s between $T_m$ and $T_g$ and an average cooling rate of 130 °C/s in this interval. For rewarming from −196 °C to −10 °C, the residence time was 1.10 s between $T_g$ and $T_m$ and an average rewarming rate of 80 °C/s. The entire 450 µm composite was thus estimated to equilibrate on a sub-2-second diffusion timescale.

For the *in situ* brain, we used a spherical model with internal conduction as the rate-limiting step. Approximating the vitrified portion of the mouse brain as a sphere of radius R ≈ 4 mm and assuming homogeneous thermal properties ($\alpha \approx 1 \times 10^{-7}\ m^2/s$, $k \approx 0.5\ W \cdot m^{-1} \cdot K^{-1}$), the first-mode analytical solution for a sphere with fixed surface temperature predicts that the center temperature evolves approximately as $T_{center}(t) = T_s + (T_0 - T_s) \cdot \exp(-t/\tau)$, with $\tau \approx R^2/(\pi^2 \alpha) \approx 16$ s as the characteristic diffusion time. For cooling from −20 °C to a −140 °C bath, this model yields a residence time of 34.7 s between $T_m$ and $T_g$ and an average central cooling rate of 2.5 °C/s. For rewarming from −140 °C to a 4 °C bath, the same model predicts an estimated residence time of 17.6 s between $T_g$ and $T_m$ and an average central rewarming rate of ~5.0 °C/s. These estimates are in reasonable agreement with the measured phantom values and meet known critical cooling and rewarming rates for VM3 (20).

**Metabolic analysis.** After cryopreservation using the vitrification protocols, the slices were incubated for 2 hours in carbogenated aCSF at RT to allow recovery. Agilent Seahorse XFe96 analyzer was used to assess oxygen consumption rate (OCR) to elucidate the mitochondrial capacity of the brain slices (21), using the Seahorse mito-stress test kit according to the manufacturer's instructions. Biopsies were then taken from the CA1 region of the hippocampal slices using 0.75 mm biopsy punches (**fig. S7**). These biopsy punches were immediately placed into individual wells of a Seahorse islet capture plate, a 96-well plate format filled with aCSF, for OCR measurements. The hippocampi were incubated at 37 °C without $CO_2$ for 45 min prior to recordings. Baseline OCR recordings were conducted to assess mitochondrial capacity. Following this, the biopsies were sequentially treated with 25 µM of the ATPase pump inhibitor oligomycin to inhibit mitochondrial oxygen consumption, then with 7.5 µM of the ionophore Carbonyl cyanide-p-trifluoromethoxyphenylhydrazone (FCCP) to determine maximal

mitochondrial capacity and proton leakage. Finally, a mixture of 5 µM rotenone and 5 µM antimycin A, inhibitors of electron transport chain complexes I and III, respectively, was added to block mitochondrial electron transport. All inhibitor concentrations were directly adopted from Underwood et al. (21).

**Light and electron microscopic analysis.** For visual inspection of slices, a Leica Ivesta 3 videostereomicroscope was used. For light microscopy to evaluate brain structure and neural morphology, the brain slices with standard CPA loading of 59% w/v, with and without vitrification, were fixated with 4% paraformaldehyde overnight at 4 °C, washed, and kept in phosphate buffered saline (PBS). Frozen sections of 30 µm were obtained and stained with cresyl violet (Nissl staining). For electron microscopy, the post-vitrification slices were fixated in 2% glutaraldehyde and 1% formaldehyde in cacodylate buffer for 24 hours, washed overnight in cacodylate buffer, postfixed with OsO4, dehydrated, and embedded in Epon (Roth, Germany). Semithin sections (1 µm) were stained with toluidine blue. Ultrathin sections (50 nm) were stained with uranyl acetate and lead citrate and viewed with a Zeiss EM 900N electron microscope.

To evaluate somatic and dendritic morphology post-vitrification, we took advantage of Slick-V Cre-YFP-expressing mice which express YFP sparsely in the hippocampal neurons (22), making it suitable to visualize and quantify spines of individual dendritic branches. For this purpose, acute hippocampal slices (350 µm) from adult YFP-labelling mice were prepared as described above. Hippocampal slices of the left hemispheres were allowed to recover in gassed aCSF at RT for 2 h before being put into 4% PFA. After 1 h incubation in aCSF (at RT), slices of the right hemisphere were subjected to the slice vitrification and rewarming protocol (60 min at -196 °C) and then were kept in aCSF for 1 h (at RT) before being immersed in 4% PFA. After 6 h fixation in PFA at 4°C, control and post-vitrification slices were washed with PBS, and imaged using a Zeiss LSM 710 (20x objective, NA 0.8 for overview images and 63x objective NA 1.4 for spine analysis) in an imaging chamber (RC-41LP, Warner Instruments). Analysis of dendritic protrusions was obtained with images of apical secondary dendrites, approximately 80 µm from the soma (23). Maximum projections of the slices were generated using Fiji software and deconvolution was performed using Truesharp software from Abberior. Spine numbers and length were quantified using the SpineJ plugin in Fiji. In total 5 slices from 3 mice were imaged and analyzed, showing no significant differences (fig. S8).

**Electrophysiological recordings in the hippocampus.** Whole-cell recordings and extracellular field potential recordings were performed in the CA1 region and the dentate gyrus (DG) of the hippocampal formation from control and treated slices, having rested 1-10 h in the incubating chamber at RT. Individual slices were transferred to a submerged chamber that was mounted on the stage of an upright microscope, and perfused with normal aCSF with 1.5 mM $MgCl_2$ and 2.5 mM $CaCl_2$ at 31 ºC. Recorded signals were filtered at 6 kHz (for action potentials) or 2 kHz (for field potentials) and sampled at 20 kHz using a Multiclamp 700B amplifier together with Digidata 1550A interface and pClamp10.7 software (Molecular Devices, Sunnyvale, CA, USA).

Field postsynaptic potentials (fPSPs) in CA1 stratum radiatum and DG middle molecular layer were recorded with a glass pipette filled with modified aCSF, in which $NaHCO_3$ was replaced by HEPES (5 mM) to avoid pH change. A concentric platinum bipolar electrode was inserted into the stratum radiatum to stimulate Schaffer collaterals (SC; **fig. S9A**), or into DG molecular layer to stimulate medial perforant path (mPP), using constant current pulses 0.1 ms wide. The input-output relationship of fPSPs was routinely determined by stepwise increase in stimulus strength from 50 to 200 µA. Short-term potentiation (STP) was examined using a brief stimulus train consisting of 5 pulses at 20 Hz. During baseline recording, strength of electrical stimuli, which were delivered at 0.1 Hz, were adjusted to elicit 30-40% of the maximum response. Long-term potentiation (LTP) of the SC-CA1 synapse was induced by means of high frequency stimulation (HFS, 100 Hz for 1s, repeated once after 20 s). LTP of the mPP-DG synapse required stronger HFS (100 Hz x 1s, repeated 4 times at 10 s intervals) to overcome robust GABAergic inhibition in

this area. fPSPs were monitored for 60 min post HFS, and data points were obtained by averaging six responses every minute. Slopes were calculated from rising phase (20-80%) of fPSPs, normalized as percentage of baseline value, and pooled across experiments of the same group.

Whole-cell current-clamp and voltage-clamp recordings were performed on visually identified cells in CA1 and DG of dorsal hippocampal slices. Patch pipettes were filled with (in mM) 135 K-gluconate, 5 HEPES, 3 $MgCl_2$, 5 EGTA, 2 $Na_2ATP$, 0.3 $Na_3GTP$, 4 NaCl (pH 7.3). Electrode resistance with internal solution in bath was 3-5 MΩ. After Giga-seal formation, whole-cell configuration was achieved by membrane rupture in voltage-clamp mode (Vh -70 mV). Hippocampal cell properties, including membrane capacitance and input resistance (Rm), were collected 2-3 min later with a 'membrane test' protocol' delivering 10 mV biphasic pulses. For current-clamp recording, Rm was re-examined after switching to current-clamp mode using hyperpolarizing pulses (5 mV). Spontaneous postsynaptic potentials (spPSPs) were collected at resting membrane potential (RMP). All potentials were corrected for liquid junction potential. To test cell excitability, depolarizing pulses (50, 100, 150 and 200 pA for 1 s) and ramps (from 0 to 100 pA within 2 s) were used to elicit action potentials (AP), with membrane potential initially held at -70 mV by injecting current. For voltage-clamp recordings, spontaneous postsynaptic currents (spPSCs) were monitored with membrane potential clamped at -80 mV (for excitatory drive) and at 0 mV (for inhibitory drive), respectively.

Electrophysiological data analysis was performed off-line with Clampfit 10.7 (Molecular Devices, CA, USA). Peak amplitude and slope (20-80% of rising phase) of evoked population responses (field PSPs) were used to quantify basic features of synaptic transmission (e.g. input-output curve), STP and LTP magnitude, respectively. For spontaneously occurring synaptic events in whole-cell recordings, the frequency of synaptic potential/current was detected. Specifically, spPSCs with voltage clamped at -80 mV or at 0 mV were used to calculate the E/I ratio (excitation/inhibition balance) in individual cells. For cell excitability, the number of APs during steps or ramps was calculated. Rheobase was determined as minimal current necessary to elicit $1^{st}$ AP during ramps. AP parameters, including threshold, rising slope, peak amplitude, half-width and fast afterhyperpolarization, were analyzed for the first AP after depolarizing pulse (100 pA; **fig. S10**). To characterize the firing behavior of APs during depolarizing steps, the adaptation ratio for AP amplitude and firing frequency was determined with repetitive AP discharges to 100 pA depolarizing pulse (**fig. S10D-E**). The adaptation ratio of AP amplitude was obtained by dividing the last AP amplitude to the first one. The interval between first and second APs was measured to determine the initial firing rate, and the interval between last 2 APs was used to define the last firing rate, respectively. AP frequency adaptation ratio was then calculated by dividing the last firing rate by the initial firing rate.

**Biocytin labelling.** In some experiments, 1% biocytin (BioTrend Chemicals GmbH, Cologne, Germany) was included in whole-cell recording solution. The recording pipette was carefully retracted at the end of the experiment, and the slice was fixed with 4% paraformaldehyde overnight at 4 °C. The slices were then washed several times with 0.1 M phosphate buffer (PBS) and kept in PBS. Blocking and permeabilization was performed by using a PBS-based blocking buffer, containing 1% bovine serum albumin, 0.5% Triton X-100 and Cy3 coupled streptavidin (Cy™3 Streptavidin, 1:1000, Jackson Immuno Research, Suffolk, UK) overnight. After PBS washing, the slices were mounted with Roti®-Mount FluorCare DAPI on cover slips. Confocal imaging was performed on a Zeiss LSM 780 confocal microscope by using 20x and 63x objectives. Fluorophores were excited at 561 nm wavelength. ZEN 2010 and ImageJ software were used for editing confocal images.

**Statistics.** Data were expressed as means ± SEM. OriginPro 2021G (OriginLab Corporation, MA, USA) was used for electrophysiological statistics and figures. Shapiro-Wilk test was used to assess normality of data distribution, and the null hypothesis was accepted when p-value was larger than 0.05. Statistical comparisons were performed using unpaired or paired Student's t-test and one-way analysis of variance (ANOVA) followed by Tukey's post-hoc test, as appropriate.

Significance was assumed for $p < 0.05$. Spearman rank-order correlation coefficient of OCR measurements was performed using the spearmanr and permutation_test function from the SciPy library (version 1.14.1) in Python (24).

**Abbreviations**

aCSF, artificial cerebrospinal fluid; AP, action potential; AQP4, aquaporin 4; BBB, blood-brain barrier; CPA, cryoprotective agent; DG, dentate gyrus; DMSO, dimethyl sulfoxide; EG, ethylene glycol; fAHP, fast afterhyperpolarization; fPSP, field postsynaptic potential; LTP, long-term potentiation; OCR, oxygen consumption rate; RMP, resting membrane potential; Rm, membrane input resistance; RT, room temperature; SC, Schaffer Collaterals; spPSP, spontaneous postsynaptic potential; STP, short-term potentiation

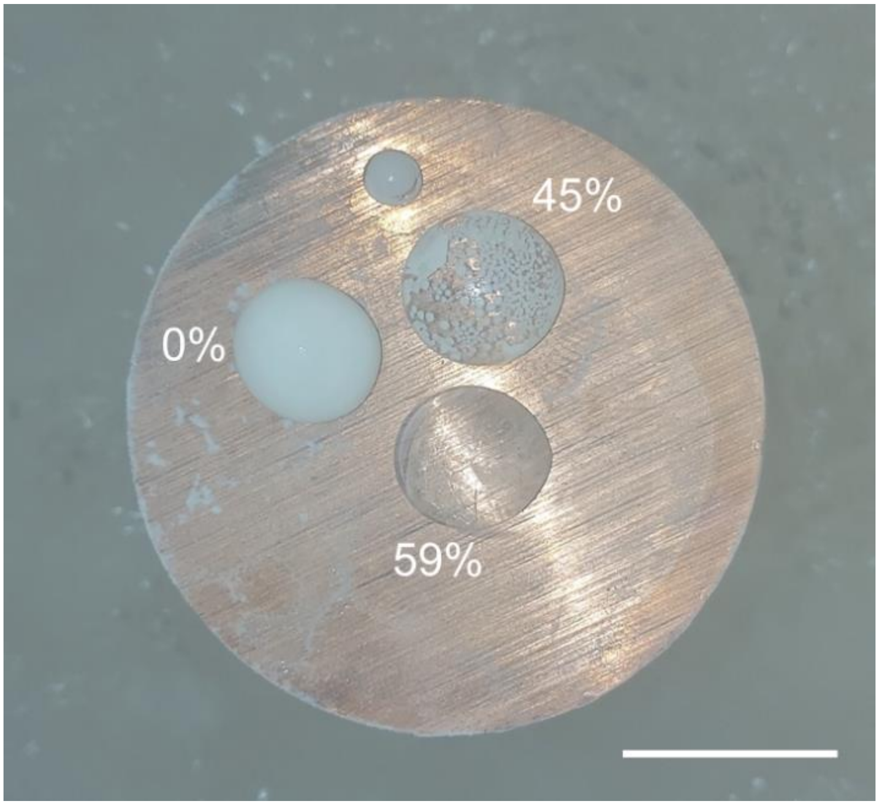


**Fig. S1. Vitrification on copper cylinder**

For directional cooling, a copper cylinder was immersed in liquid nitrogen (view from above). 1 ml droplets of LM5 carrier solution with 0% w/v V3 shows complete crystallization, 45% w/v partially crystalline, partially vitreous, 59% is completely vitreous. No cracking occurred during directional cooling from below. Scale bar 1 cm.

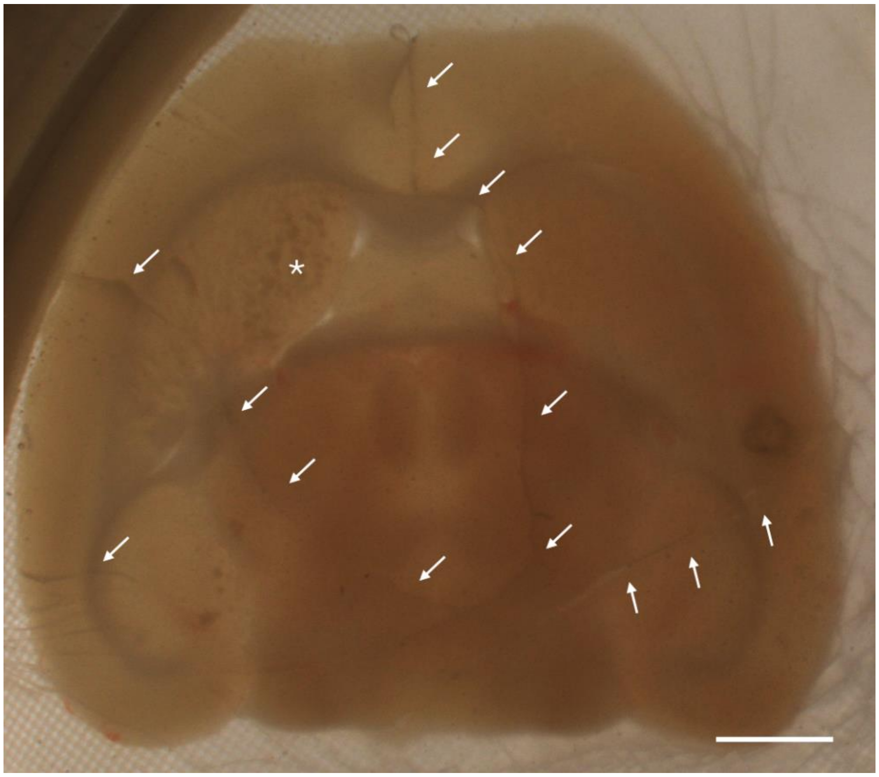

**Fig. S2. Damage from cracking due to thermomechanical stress and crystallization**
Destruction of brain slices due to cracking during direct immersion in liquid nitrogen. Tissue cracks are visible at cryogenic temperature and marked with white arrows. A limited degree of cooling phase crystallization is visible in the striatum on the left side of the image (asterisk). A limited degree of diffuse rewarming phase crystallization is visible on the right side of the image (diffuse darkening). Stereo microscopy with -160 °C isopentane covering. Lighting from behind. Image contrast enhanced by 42%. Scale bar 1 mm.

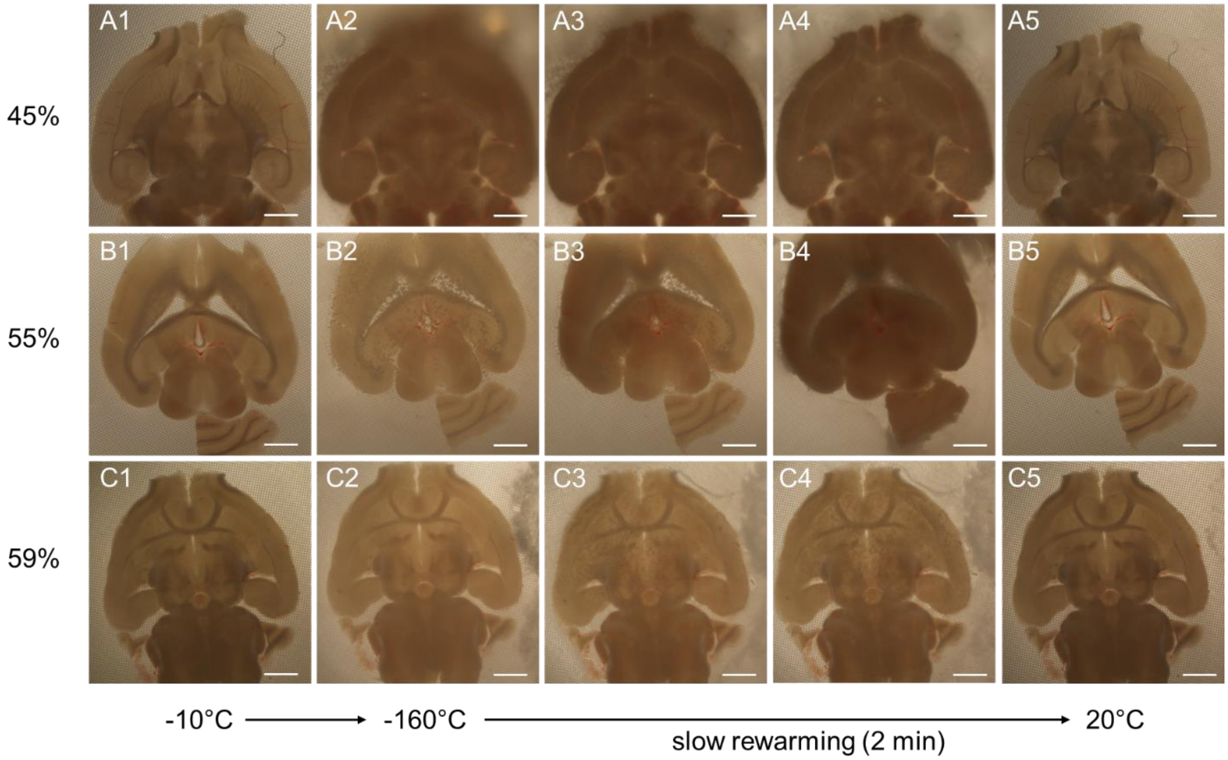


**Fig. S3. Cooling and rewarming phase crystallization of brain slices.**
Destruction of brain slices due to cooling and rewarming phase crystallization during slow rewarming. Brain slices were incubated at -10 °C for 2h in target concentrations of 45% (**A1**)-(**A5**), 55% (**B1**)-(**B5**), and 59% w/v V3 (**C1**)-(**C5**), followed by brief immersion in 65% w/v V3. (**A1**), (**B1**), (**C1**) show slices at the end of incubation. (**A2**), (**B2**), (**C2**) show slices at cryogenic temperature after cooling on a -196 °C copper cylinder and -160 °C Isopentane covering. (**A2**) shows extensive, (**B2**) slight, (**C2**) no cooling phase crystallization. (**A3**) and (**A4**) show further loss of transparency during rewarming, which returns to baseline transparency after completed rewarming to RT in (**A5**). (**B3**) and (**B4**) show extensive rewarming phase crystallization with loss of transparency, which returns to baseline transparency after rewarming to RT in (**B5**). (**C3**) and (**C4**) show slight rewarming phase crystallization and return to baseline in (**C5**) at RT. Rewarming phase is recorded in Movie S1, S5, S7. Stereo microscopy with -160 °C isopentane covering. Lighting from behind. Image contrast enhanced by 42%. Scale bars 1 mm.

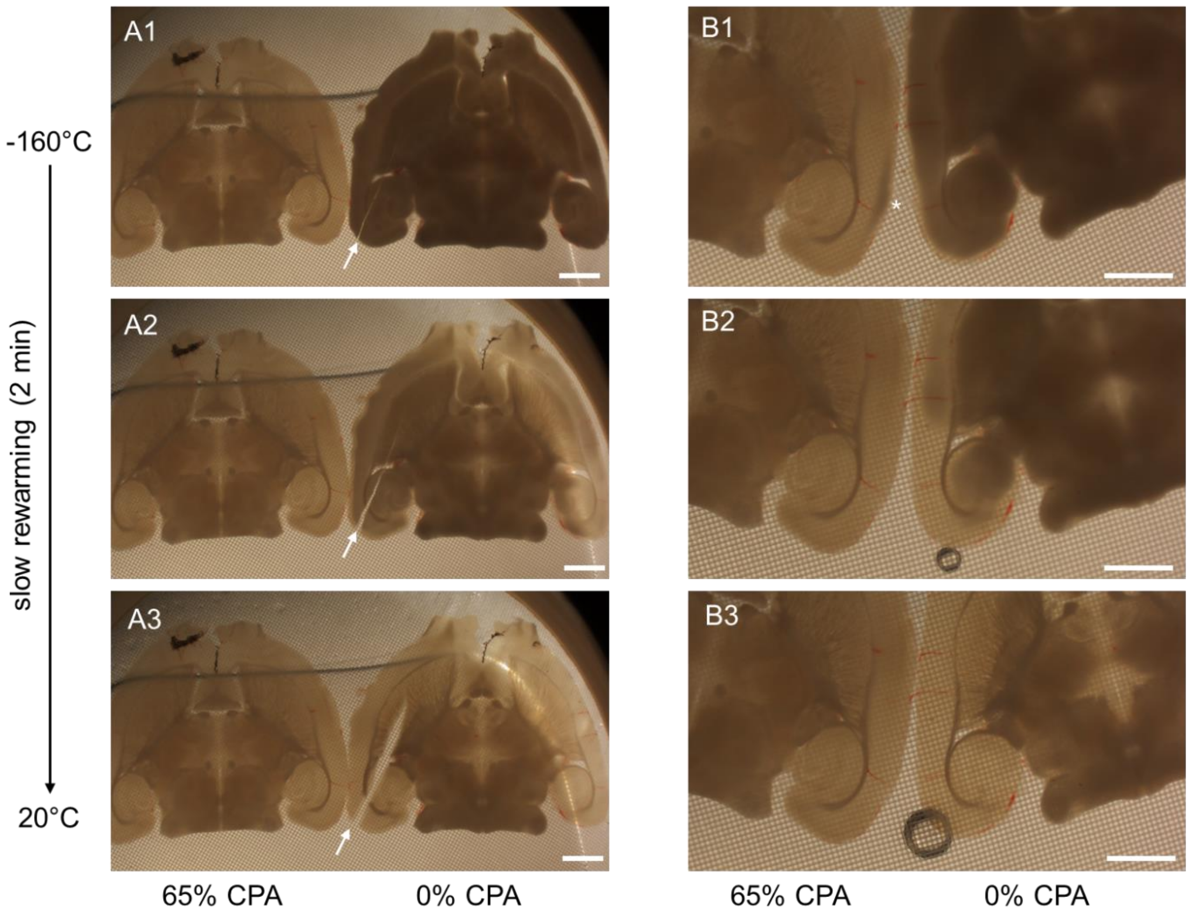


**Fig. S4. Stable vitrification vs. crystallization and cracking of brain slices**
Comparison of stable vitrification (left) vs. crystallization (right) of brain slices. The left slice was incubated at -10 °C for 2h in a target concentrations of 65% w/v V3, the right slice was incubated in LM5 carrier solution only. (**A1**) and (**B1**) show slices at cryogenic temperature after cooling on a -196 °C copper cylinder and -160 °C Isopentane covering with loss of transparency due to crystallization in the right slice. Transparency returns gradually (**A2**), (**B2**) during rewarming to RT (**A3**), (**B3**), while the vitrified is rewarming without crystallization. (**A1**)-(**A3**) show a crack from thermomechanical stress in the crystallized slice (Arrows). (**B1**) shows crystallization in the vitrified slice due to loss of CPA (asterisk). The rewarming phase is recorded in Movie S12. Stereo microscopy with -160 °C isopentane covering. Lighting from behind. Image contrast enhanced by 42%. Scale bars 1 mm.

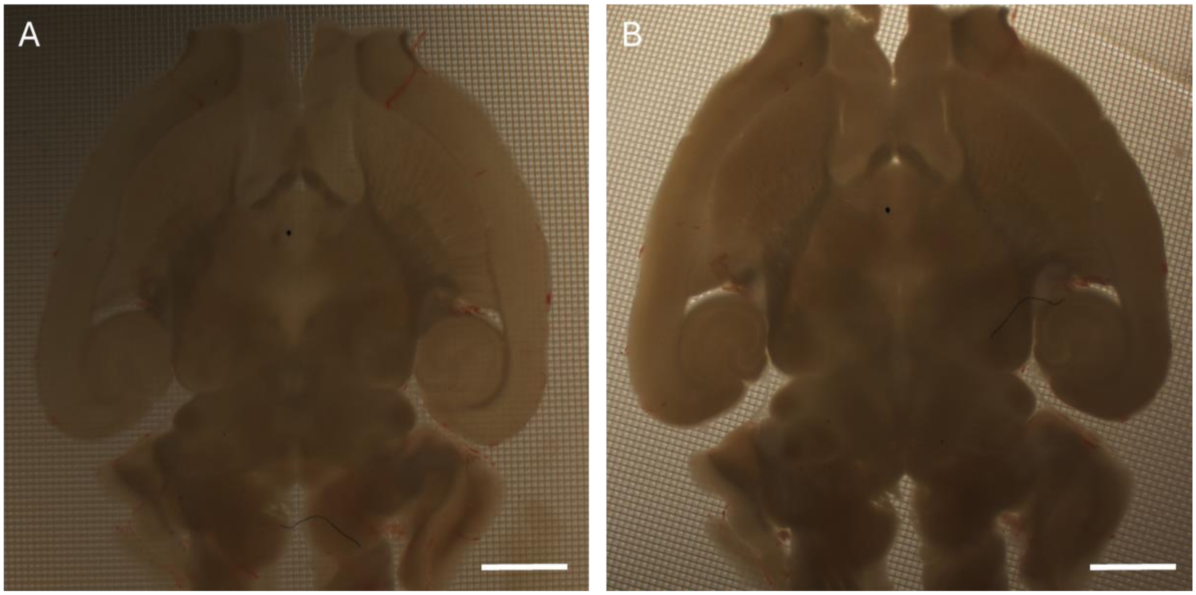


**Fig. S5. Swelling and loss of transparency during CPA unloading.**
Comparison of the same brain slice during optimized vitrification protocol. (**A**): Slice after incubation step with 59% w/v V3. (**B**): Slice after incubation step with 16% w/v V3 and added mannitol shows loss of transparency and swelling. The area of slice is increased by 8% in (**B**) compared to (**A**). Lighting from behind. Image contrast enhanced by 42%. Scale bars 1 mm.

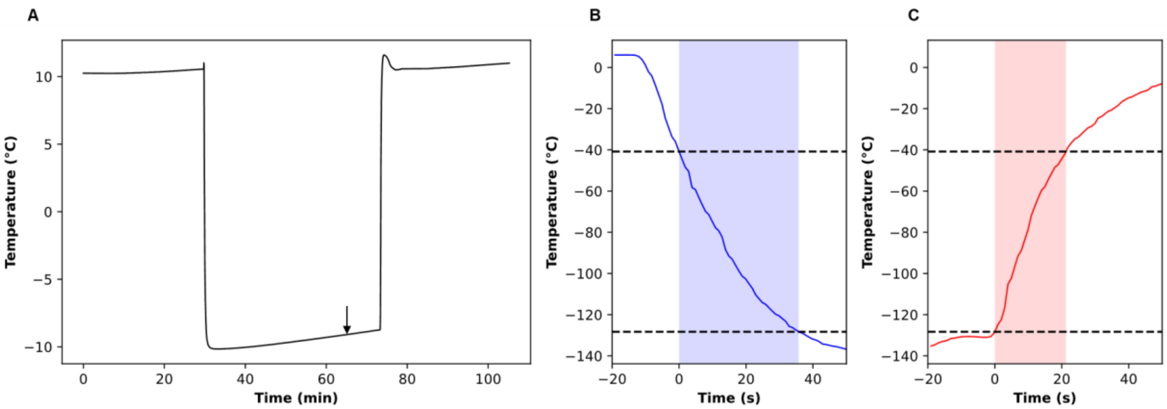


**Fig. S6. Temperature recordings.**
(**A**) Sample temperature traces during gradual CPA loading and washout. Target temperatures of 10 °C and −10 °C were maintained within ±1 °C by manual adjustment of the respective water baths (addition of blue ice for 10 °C, dry ice for −10 °C). The time point used for vitrification/rewarming is indicated by an arrow. (**B**) Cooling rate measurement using a 0.5 ml phantom containing 327 mg V3 vitrification solution with a centrally placed thermocouple. Transfer from a 4 °C bath to a −140 °C isopentane bath yielded a transit time of 35.7 s (blue shaded area) through the metastable region between $T_m$ = −40.8 °C (upper dashed line) and $T_g$ = −128.3 °C (lower dashed line), corresponding to an average cooling rate of 2.45 °C/s. (**C**) Rewarming rate measurement of the same phantom during transfer from −140 °C isopentane to a 4 °C V3 bath. The interval between $T_g$ and $T_m$ was traversed in 21.3 s (red shaded area), corresponding to an average rewarming rate of 4.11 °C/s.

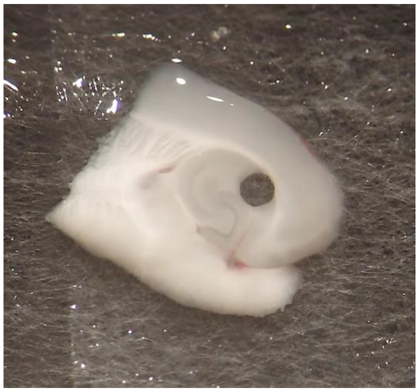

**Fig. S7. Metabolic analysis, biopsy location**
Representative image for location of biopsies for metabolic analysis that were then taken from the CA1 region of the hippocampal slices using 0.75 mm biopsy punches.

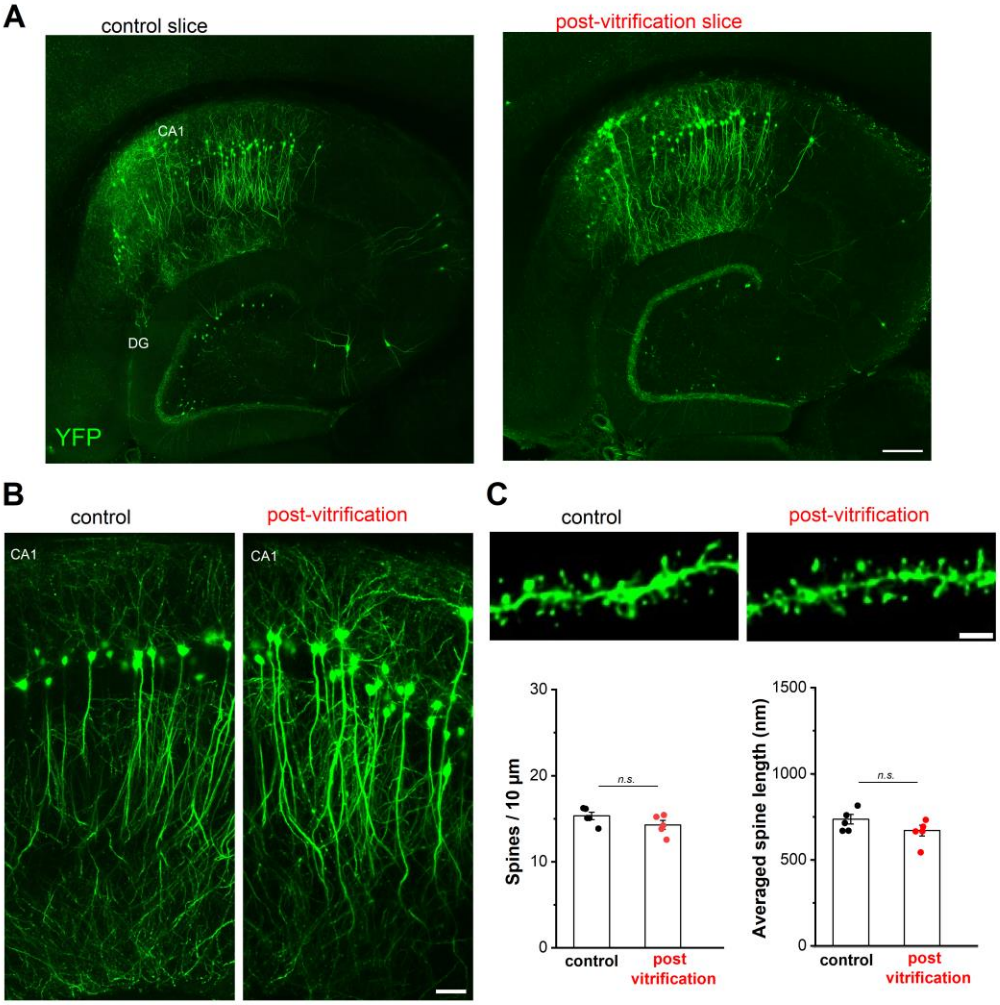


**Fig. S8. Preservation of hippocampal cell architecture and dendritic spine morphology after slice vitrification.**
**(A)** Low-magnification confocal images of YFP-expressing hippocampal cells in acute horizontal slices from Slick-V Cre-YFP mice. Control slice (left) and post-vitrification slice (right; 1h at −196 °C, followed by rewarming and 1 h incubation in aCSF) show comparable somatic and dendritic labeling across CA1 and DG. **(B)** Higher-magnification view of CA1 pyramidal cells reveals intact somatic morphology and apical dendritic organization in both control and post-vitrification slices. **(C)** Representative images of apical secondary dendrites (~80 µm from the soma) from control (left) and post-vitrification (right) slices used for spine quantification. Summarized plots for spine density and spine length show no significant differences between groups (n = 5 slices from 3 mice). Scale bars: 200 µm (**A**), 50 µm (**B**); 2 µm (**C**).

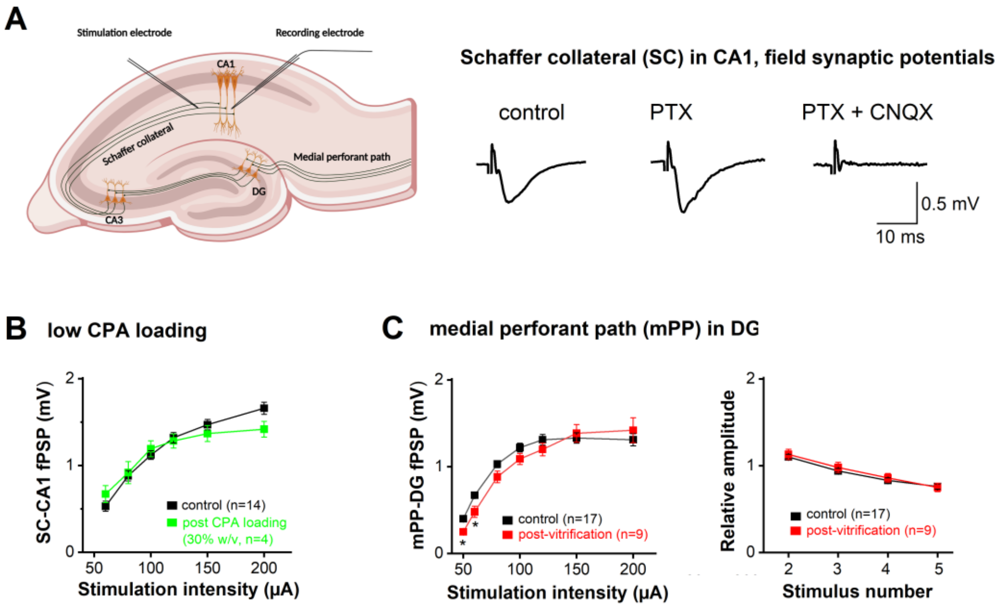


**Fig. S9. Properties of synaptic transmission derived from extracellular field recordings in area CA1 and dentate gyrus.**
(**A**) Schematic drawing of canonical tri-synaptic circuit within a murine hippocampal slice showing location of stimulating and recording electrodes to examine fPSPs at the SC-CA1 synapse. Raw voltage traces on the right depict fPSPs from a post-vitrification slice (10 min at -196 °C and 4 h post rewarming) in normal bath solution (control), in the presence of the $GABA_A$ receptor antagonist picrotoxin (PTX, 100 µM), and in the presence of the AMPA receptor antagonist CNQX (40 µM); stimulus intensity was 70 µA. Drawing was created in BioRender. German, A. (2025) https://BioRender.com/b67t366 (**B**) Effect of CPA loading with 30% w/v on I-O curves from SC-CA1 synapse. (**C**) Slight downward shift of I-O curve from mPP-GC synapse after vitrification when compared to untreated controls (left panel). In contrast, STP remained unaffected after vitrification (right panel). * $p < 0.05$

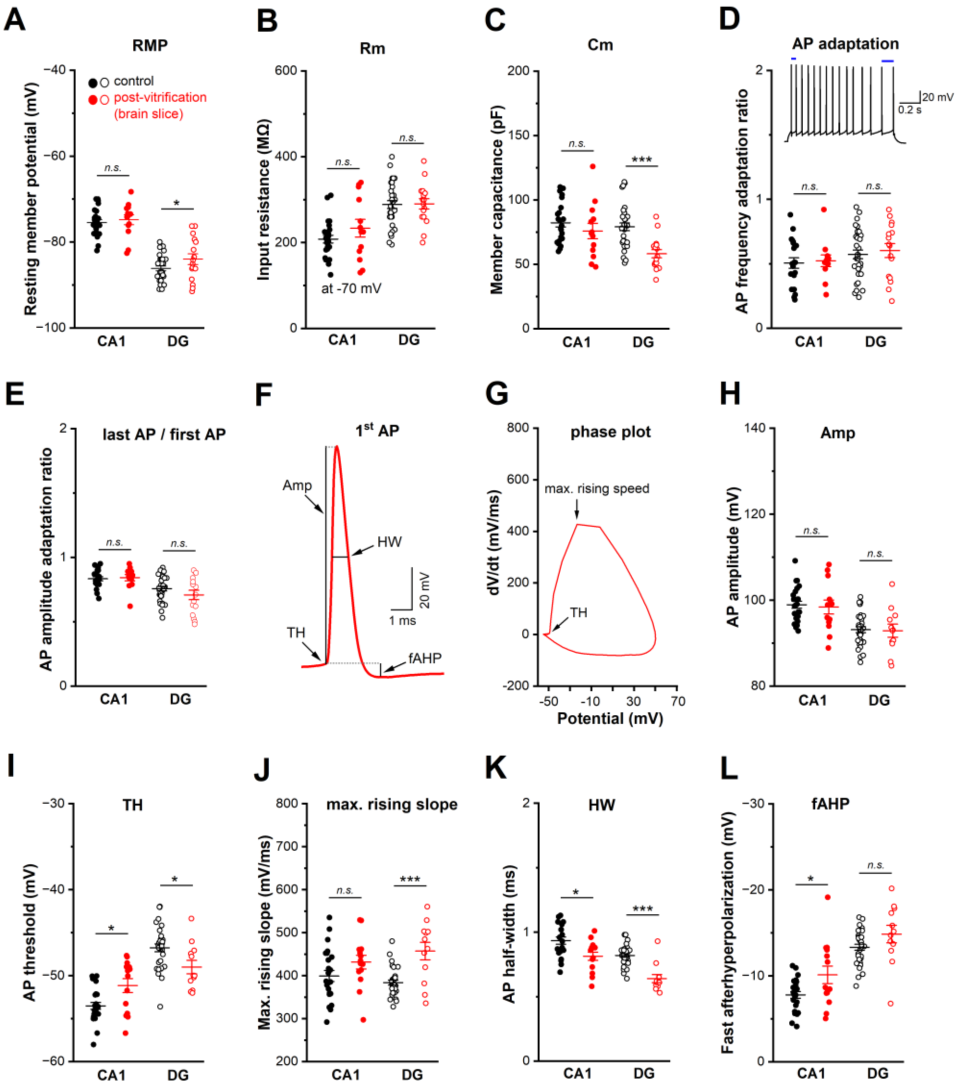


**Fig. S10. Passive membrane properties and action potentials in hippocampal principal cells post slice vitrification.**
(**A-C**) Summary of resting membrane potential (RMP, **A**), membrane input resistance ($R_m$, **B**) and capacitance ($C_m$, **C**) in hippocampal cells from brain slices with and without vitrification. (**D-E**) Characterization of action potential (AP) adaptation. The adaptation ratio for AP firing frequency (**D**) and peak amplitude (**E**) was determined with repetitive AP discharge to 100 pA depolarizing pulse. The intervals between first 2 APs and between last 2 APs (as indicated by *blue lines and arrows above the inserted* control trace) were used to define the initial and the last firing rate, respectively (**D**). The adaptation ratio of AP amplitude was obtained by dividing the last AP amplitude to the first one (**E**). (**F-G**) Single AP waveform and phase plot from CA1 pyramidal cell from a post-vitrification slice (1 day at -150 °C and 6 h post rewarming in normal bath solution) demonstrating how parameters were defined. (**H-L**) Individual data points for peak amplitude (**H**), voltage threshold (**I**), maximum rising slope (**J**), half-width (**K**) and fast afterhyperpolarization (fAHP; **L**) of evoked action potential. Statistical comparisons were performed using an unpaired, two-tailed t-test. n.s. not significant; * $p < 0.05$; *** $p < 0.001$.

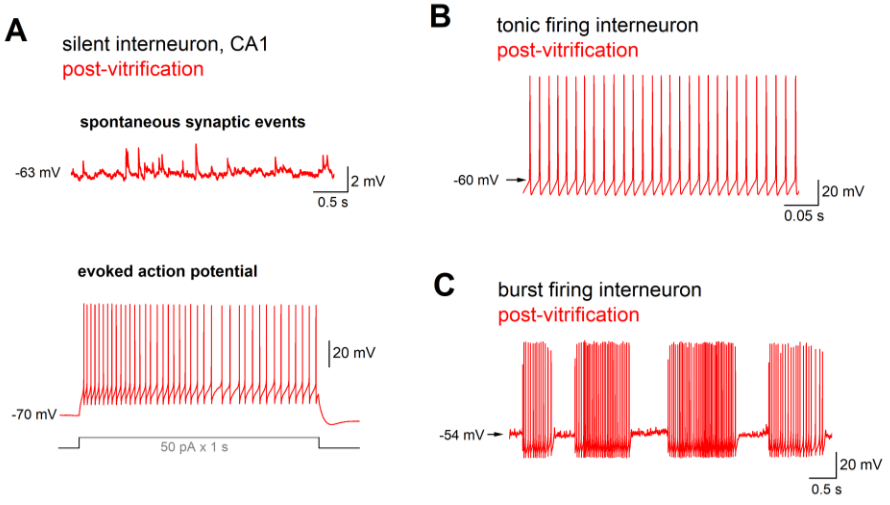


**Fig. S11. Evidence for neurophysiologically sound CA1 interneurons after vitrification**
Whole-cell current-clamp recordings from interneurons whose somata were located within or next to CA1 pyramidal cell layer, all from slices post-vitrification. (**A**) Silent interneuron receiving spontaneous synaptic input at rest (-63 mV), and discharging action potentials upon depolarization (50 pA for 1 s; initially held at -70 mV). (**B-C**) Examples of two electrophysiologically distinct interneurons exhibiting spontaneous action potential firing at rest, either in a tonic fashion (**B**), or in burst-like mode (**C**).

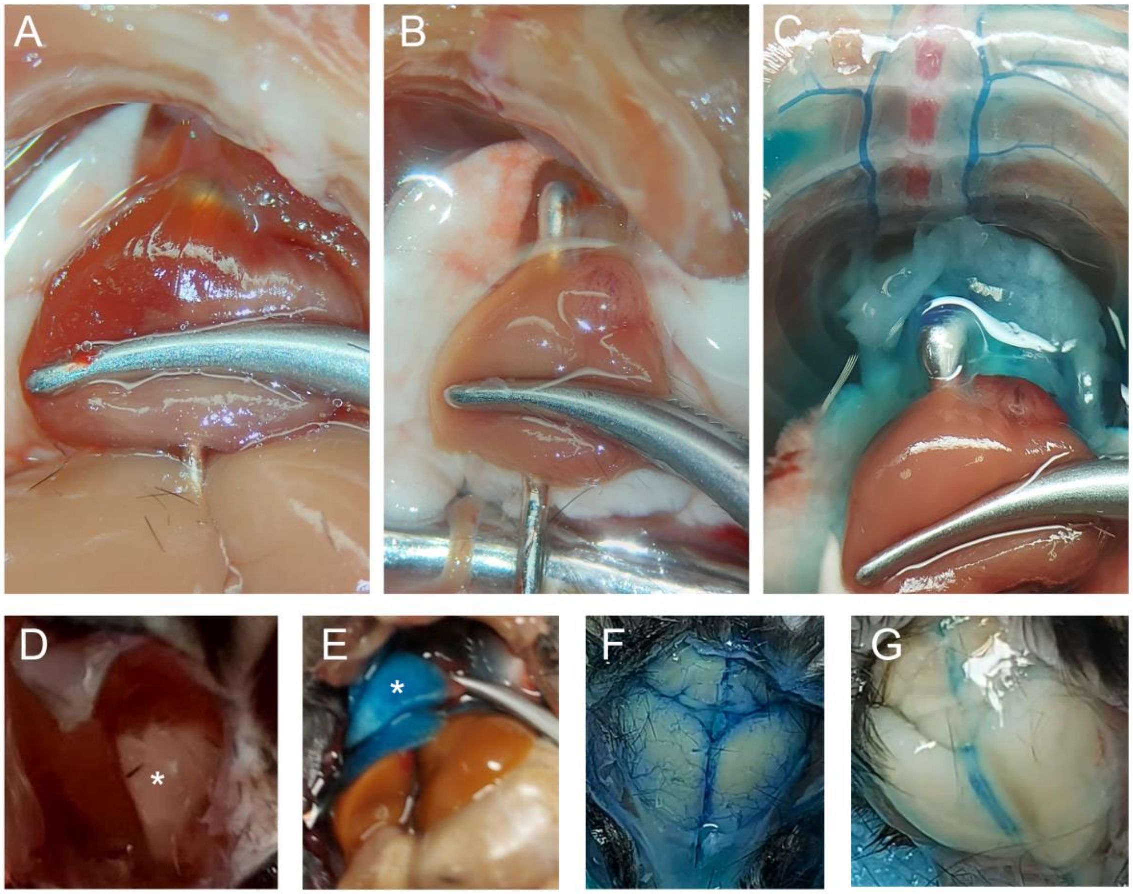


**Fig. S12. Perfusion strategy for cephalothoracic specimens and failure modes.** The button cannula was inserted via the left ventricle and secured with small hemostatic forceps (**A-C**). For perfusion steps prior to vitrification, the equator of the button cannula was not advanced beyond the atrioventricular plane (**A**). Post-rewarming, the button was fully advanced into the ascending aorta (**B**). Quality of perfusion was monitored visually at the internal thoracic arteries via addition of 0.01% w/v methylene blue to LM5 during interleaved equilibration and to the initial 10% dextran washout perfusate (**C**). Several failure modes of the protocol were visually detectable (**D-G**). Apparent crystallization of the left cerebral hemisphere (asterisk) vs. apparent vitrification of the right brain hemisphere after immersion in cryogenic isopentane (**D**). Pulmonary edema (asterisk) due to reflux in the pulmonary veins during CPA washout via addition of 0.01% w/v methylene blue to the initial 10% dextran washout perfusate (**E**). Successful cerebral perfusion was confirmed via staining of small pial vessels with methylene blue, as present in (**F**). Absence of this cerebral vessel staining indicated insufficient cerebral perfusion. Cerebral edema was detectable via bulging from the skull base and tissue whitening (G).

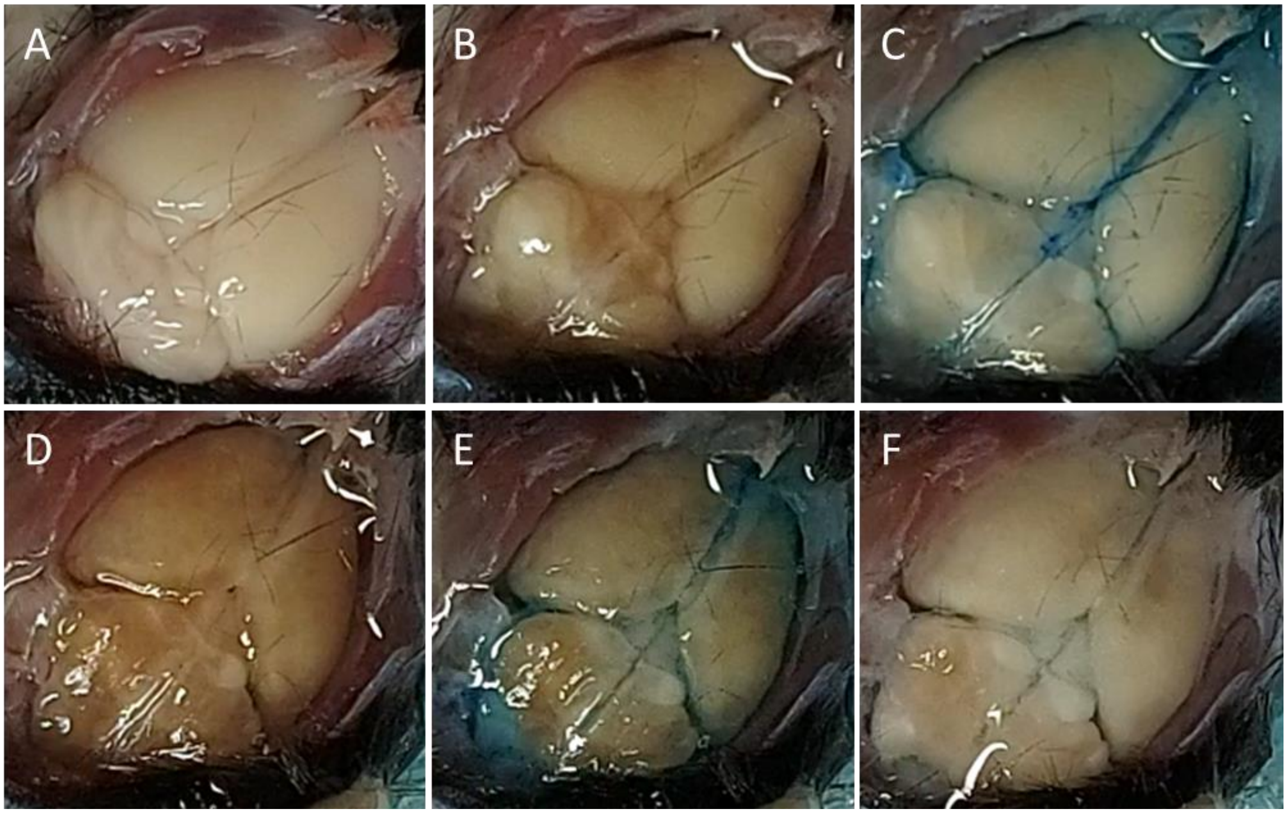


**Fig. S13. Macroscopic changes of the same brain during interleaved equilibration and hyperoncotic washout.**
The brain was exposed during the entire perfusion protocol via craniectomy after incision along the sagittal suture from the foramen magnum (**A-F**), showing the non-dehydrated native initial state (**A**), the dehydrated state after the first 8-minute CPA-perfusion step (**B**), the rehydrated state after the 3-minute LM5-perfusion step (**C**), the fully CPA-loaded state after the 25-minute CPA-perfusion step (**D**), the state during the first hyperoncotic washout step (**E**), the normalization of brain volume after completion of the hyperoncotic washout (**F**). Note that due to craniectomy in the non-dehydrated native state (**A**), median-sagittal damage to the cerebral vasculature and parenchyma is evident in (**D-F**). This could be prevented by delaying craniectomy to state (**B**).

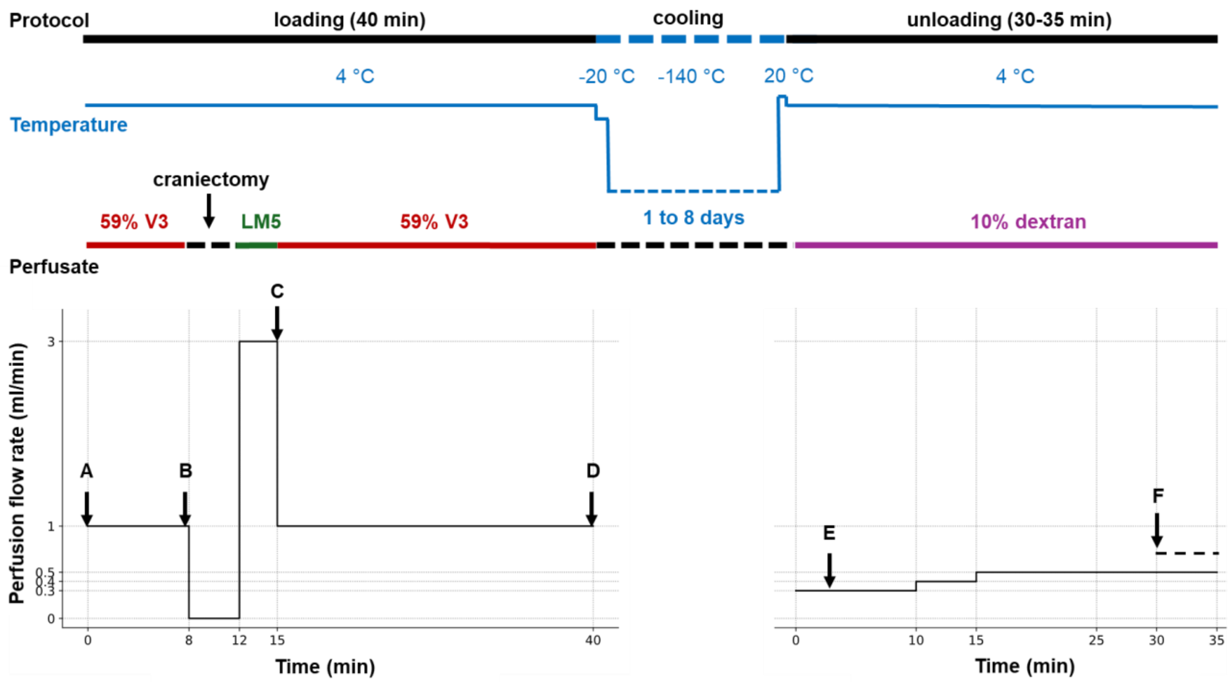


**Fig. S14. Protocol for cerebral vitrification *in situ*.**
Time course of vitrification protocol, with varied temperature (top), perfusate (middle) and perfusion flow rate (bottom). Arrows indicate when images (**A-F**) in Fig. S13 were taken. Dotted line indicates when perfusion was terminated.

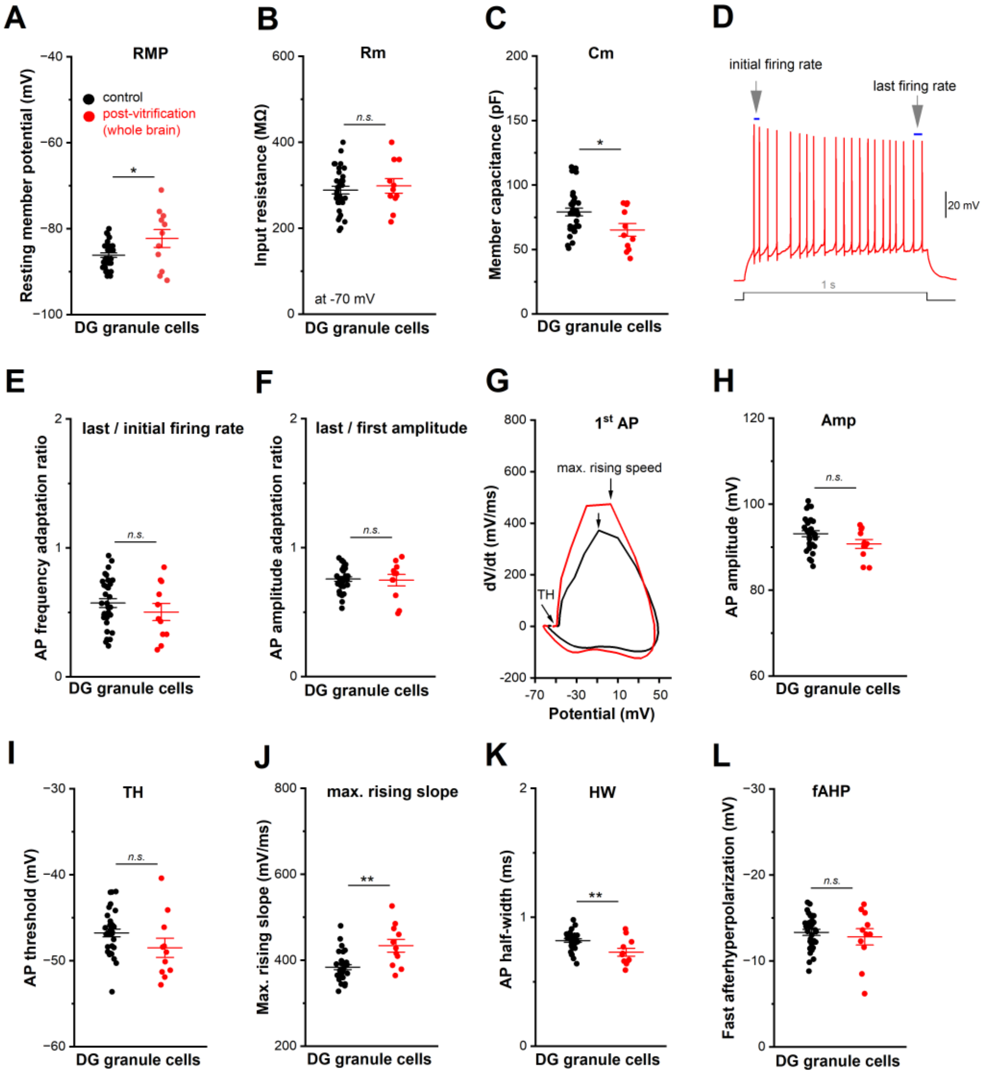


**Fig. S15. Properties of dentate gyrus granule cells after *in situ* brain vitrification.** (**A-C**) Summary of RMP, $R_m$ and $C_m$ in DG granule cells post *in situ* vitrification. (**D**) Voltage trace from a granule cell from a brain after vitrification (8 days at -150 °C and 5 h post rewarming), illustrating how the initial and the last firing rate were determined for characterization of AP frequency adaptation. (**E-F**) Adaptation ratio for AP frequency (**E**) and amplitude (**F**), obtained with repetitive discharge (around 20 APs) in response to depolarization step (pulse duration 1 s). (**G**) Superimposed phase plots of granule cell APs from a control brain (black plot) and a post-vitrification brain (red plot). (**H-L**) Summary of peak amplitude (**H**), voltage threshold (**I**), maximum rising slope (**J**), half-width (**K**) and fAHP (**L**) of evoked AP. Statistical comparisons were performed using an unpaired, two-tailed t-test. *n.s.* not significant; * $p < 0.05$; ** $p < 0.01$.

**Table S1.**
**Carrier solution LM5.**

| chemical | concentration |
|---|---|
| glucose | 90 mM |
| mannitol | 45 mM |
| lactose | 45 mM |
| KCl | 8.2 mM |
| $K_2$HPO4 | 7.2 mM |
| glutathione | 5 mM |
| adenine | 1 mM |
| $NaHCO_3$ | 10 mM |

**Table S2.**
**Modified standard vitrification solution (59% w/v V3 = 8.42 M permeable CPA).**

| chemical | concentration |
| --- | --- |
| dimethyl sulfoxide (DMSO) | 22.3% w/v |
| ethylene glycol (EG) | 16.84% w/v |
| formamide | 12.86% w/v |
| polyvinylpyrrolidone K12 | 7% w/v |

**Table S3.**
**Optimized slice vitrification protocol.**

| concentration V3 | concentration mannitol | duration | temperature |
|---|---|---|---|
| *Slicing in sucrose-aCSF* | | 20 min | 4 °C |
| *Incubation in sucrose-aCSF* | | 10 min | 35 °C |
| *Incubation in aCSF* | | 30 min | 20 °C |
| 0% | 45 mM | 2 min | 10 °C |
| 2% | 45 mM | 2 min | 10 °C |
| 4% | 45 mM | 2 min | 10 °C |
| 8% | 45 mM | 2 min | 10 °C |
| 16% | 45 mM | 2 min | 10 °C |
| 30% | 45 mM | 5 min | 10 °C |
| 45% | 45 mM | 2 min | -10 °C |
| 59% | 45 mM | 5 min | -10 °C |
| 59% | 45 mM | 5 min | -10 °C |
| 59% | 45 mM | 5 min | -10 °C |
| *Liquid nitrogen* | | >10 min | -196 °C |
| 52% | 45 mM | 20 s | -10 °C |
| 45% | 345 mM | 2 min | -10 °C |
| 30% | 345 mM | 5 min | -10 °C |
| 16% | 345 mM | 10 min | 10 °C |
| 8% | 345 mM | 3 min | 10 °C |
| 4% | 345 mM | 3 min | 10 °C |
| 2% | 345 mM | 3 min | 10 °C |
| 0% | 345 mM | 10 min | 10 °C |
| 0% | 45 mM | 10 min | 10 °C |
| *Incubation in aCSF* | | >30 min | 20 °C |

**Table S4.**
**Protocol for cerebral vitrification *in situ*.**

| Perfusate | Flow rate | duration | temperature |
|---|---|---|---|
| *Thoracotomy and incision of the right atrium* | | 4 min | 36 °C |
| PBS | 10 ml/min | 2 min | 4 °C |
| *Aortic cannulation of cephalothoracic specimen* | | 2 min | 4 °C |
| 59% V3 | 1 ml/min | 8 min | 4 °C |
| *Craniectomy* | | 4 min | 4°C |
| LM5 + 0.01% methylene blue | 3 ml/min | 3 min | 4 °C |
| 59% V3 | 1 ml/min | 25 min | 4 °C |
| *Immersion in 65% V3* | | 2 min | -20 °C |
| *Immersion in isopentane* | | >1 d | -140 °C |
| *Stirring in 65% V3* | | 1 min | 20 °C |
| *Immersion in 59% V3* | | 5 min | 4 °C |
| 10% dextran + 0.01% methylene blue | 0.3 ml/min | 10 min | 4 °C |
| 10% dextran | 0.4 ml/min | 5 min | 4 °C |
| 10% dextran | 0.5 ml/min | 10 min | 4 °C |
| *Continue perfusion until normal brain size* | | 5-10 min | 4 °C |
| *Brain extraction and immersion in LM5* | | 5 min | 4 °C |
| *Slicing in sucrose-aCSF* | | 20 min | 4 °C |
| *Incubation in aCSF* | | 1 h | 20 °C |
| *Incubation in sucrose-aCSF* | | 10 min | 35°C |
| *Incubation in aCSF* | | >30 min | 20 °C |

**Table S5.**
**Failure modes of cerebral vitrification *in situ*.**

| Outcome of iteration | Count |
|---|---|
| Aortic rupture during first cannulation | 1 |
| Brain volume normalization during CPA loading (no visual gap between brain and skull base) | 2 |
| Apparent crystallization during cooling | 1 |
| Aortic rupture during second cannulation | 3 |
| Reflux in pulmonary circulation during dextran perfusion | 2 |
| Insufficient cerebral perfusion/volume normalization | 2 |
| Cerebral edema during CPA washout | 2 |
| Non-recordability (Electrophysiology and OCR) | 7 |
| Recordability (Electrophysiology and OCR) | 10 |

**Table S6.**
**Supply of chemicals used for vitrification protocol and metabolic analysis.**

| Chemical | Supplier | Catalog / ID no. |
|---|---|---|
| 2-Methylbutane | Carl Roth | 3927.2 |
| Acetazolamide | Carl Roth | 59-66-5 |
| Adenine | Sigma-Aldrich | A8626-25G |
| alpha-Lactose monohydrate | Carl Roth | 8921.1 |
| Antimycin A | Sigma-Aldrich / Merck | A8674 |
| AqB013 | Focus Biomolecules | 10-5723 |
| D(+)-Glucose | Sigma-Aldrich | G7528-1KG |
| D(-)-Mannitol | Carl Roth | 8883.2 |
| Dextran 500 | Carl Roth | 9219.1 |
| Dextran 70 | Carl Roth | 9228.2 |
| Dimethyl sulfoxide (DMSO) | Carl Roth | 7029.4 |
| Dipotassium hydrogen phosphate trihydrate | Carl Roth | 6878.2 |
| EDTA | PanReac AppliChem | A5097,0500 |
| EGTA | Sigma-Aldrich | E3889 |
| Ethylene glycol | Carl Roth | 6881.4 |
| FCCP | Cayman | 15218 |
| Formamide | Carl Roth | 4095.3 |
| Glutathione | Sigma-Aldrich | 1.04090.0050 |
| Oligomycin | Sigma-Aldrich / Merck | 495455 |
| Papain | Worthington Biochemical | LK003176 |
| PBS buffer | PanReac AppliChem | A0965,9010 |
| Polyvinylpyrrolidone (PVP) K12 | Carl Roth | 7598.1 |
| Potassium chloride | Merck | 1.04936.1000 |
| Rotenone | SCBT | sc-203242 |
| SDS ultra pure | Carl Roth | 2326.1 |
| Sodium hydrogen carbonate | Merck | 6329 |
| SonoVue | Bracco | n.a. |
| Supercool X-1000 | 21st Century Medicine | n.a. |
| Supercool Z-1000 | 21st Century Medicine | n.a. |
| TC AQP1 | Hycultec | HY-110200 |
| Tetraethylammonium | Carl Roth | 1185-59-7 |
| TGN-020 | InvivoChem | HY-W008574 |
| TRIS-HCl buffer | Carl Roth | 9090.1 |
| Trypsin/EDTA | PAN-Biotech | P10-023100 |

**Table S7.**
**Action potential (AP) parameters of mouse hippocampal principal cells post-vitrification**

| **Hippocampal cell properties** | **control**<br><br>**CA1** (n=24) | **slice post-vitrification CA1** (n=13) | **control**<br><br>**DG** (n=32) | **slice post-vitrification DG** (n=16) | ***in situ* brain post-vitrification DG** (n=11) |
|---|---|---|---|---|---|
| resting membrane potential (mV) | -74.66±0.66 | -74.82±1.12 | -85.97±0.53 | -83.17±1.43* | -82.15±1.98* |
| input resistance (MΩ) | 203.42±8.77 | 233.46±20.63 | 290.00±8.47 | 290.31±12.14 | 289.58±18.56 |
| capacitance (pF) | 82.13±3.09 | 75.84±6.01 | 75.65±3.12 | 58.18±3.17*** | 64.75±4.54* |
| AP peak amplitude (mV) | 98.94±0.83 | 98.71±1.54 | 93.13±0.68 | 92.91±1.52 | 90.07±1.03 |
| AP threshold (mV) | -53.51±0.40 | -51.42±0.83* | -46.77±0.46 | -49.00±0.77* | -48.50±1.12 |
| AP max. rise (mV/ms) | 399.11±12.78 | 429.96±17.20 | 383.94±8.33 | 457.24±20.36*** | 433.90±14.87** |
| AP half-width (ms) | 0.94±0.03 | 0.83±0.03* | 0.82±0.01 | 0.64±0.03*** | 0.73±0.03** |
| AP fAHP (mV) | -7.79±0.39 | -9.87±1.07* | -13.32±0.35 | -14.69±0.98 | -12.80±0.94 |

Statistical analysis was performed using unpaired, two-tailed Student's t-test. *$p < 0.05$, **$p < 0.01$ significant difference from respective control value.

**Movie S1 (separate file). Slow rewarming 45%**
Slow rewarming of brain slice from fig. S4A after 45% w/v V3 loading. Complete crystallization during cooling phase. Stereo microscopy with -160 °C isopentane covering. Lighting from behind. Mesh size 74 µm.
**Movie S2 (separate file). Slow rewarming 52%**
Slow rewarming of brain slice after 52% w/v V3 loading. Near-complete crystallization during cooling phase. Complete crystallization during rewarming phase. Stereo microscopy with -160 °C isopentane covering. Lighting from behind. Mesh size 74 µm.
**Movie S3 (separate file). Slow rewarming 53%**
Slow rewarming of brain slice after 53% w/v V3 loading. Partial crystallization during cooling phase. Complete crystallization during rewarming phase. Stereo microscopy with -160 °C isopentane covering. Lighting from behind. Mesh size 74 µm.
**Movie S4 (separate file). Slow rewarming 54%**
Slow rewarming of brain slice after 54% w/v V3 loading. Partial crystallization during cooling phase. Complete crystallization during rewarming phase. Stereo microscopy with -160 °C isopentane covering. Lighting from behind. Mesh size 74 µm.
**Movie S5 (separate file). Slow rewarming 55%**
Slow rewarming of brain slice from fig. S4B after 55% w/v V3 loading. Partial crystallization during cooling phase. Complete crystallization during rewarming phase. Stereo microscopy with -160 °C isopentane covering. Lighting from behind. Mesh size 74 µm.
**Movie S6 (separate file). Slow rewarming 56%**
Slow rewarming of brain slice after 56% w/v V3 loading. Partial crystallization during cooling phase. Complete crystallization during rewarming phase. Stereo microscopy with -160 °C isopentane covering. Lighting from behind. Mesh size 74 µm.
**Movie S7 (separate file). Slow rewarming 59%**
Slow rewarming of brain slice from fig. S4C after 59% w/v V3 loading. Partial crystallization during rewarming phase. Stereo microscopy with -160 °C isopentane covering. Lighting from behind. Mesh size 74 µm.
**Movie S8 (separate file). Rapid rewarming 56%**
Rapid rewarming of brain slice after 56% w/v V3 loading by covering with 52% w/v V3 solution at -10 °C. Obvious crystallization from cooling phase. Stereo microscopy. Lighting from behind. Mesh size 74 µm.
**Movie S9 (separate file). Rapid rewarming 57%**
Rapid rewarming of brain slice after 57% w/v V3 loading by covering with 52% w/v V3 solution at -10 °C. Slight crystallization from cooling phase. Stereo microscopy. Lighting from behind. Mesh size 74 µm.
**Movie S10 (separate file). Rapid rewarming 58%**
Rapid rewarming of brain slice after 58% w/v V3 loading by covering with 65% w/v V3 solution at -10 °C. No crystallization from cooling phase. Stereo microscopy. Lighting from behind. Mesh size 74 µm.
**Movie S11 (separate file). Rapid rewarming 59%**
Rapid rewarming of brain slice after 59% w/v V3 loading by covering with 65% w/v V3 solution at -10 °C. No crystallization from cooling phase. Stereo microscopy. Lighting from behind. Mesh size 74 µm.
**Movie S12 (separate file). Slow rewarming 65% vs 0%**
Slow rewarming of brain slices from fig. S5A after 65% w/v V3 loading (left) and 0% loading (right). No evidence for rewarming phase crystallization in the vitrified slice. Stereo microscopy with -160 °C isopentane covering. Lighting from behind. Mesh size 74 µm.

## SI References


1. F. Zheng *et al.*, Activin tunes GABAergic neurotransmission and modulates anxiety-like behavior. *Mol Psychiatry* **14**, 332-346 (2009).
2. Y. Pichugin, www.cryonics.org/research/blood-brain-barrier-preliminary-patent-application-disclosure. (2007).

3. G. M. Fahy *et al.*, Cryopreservation of organs by vitrification: perspectives and recent advances. *Cryobiology* **48**, 157-178 (2004).
4. Y. Pichugin, G. M. Fahy, R. Morin, Cryopreservation of rat hippocampal slices by vitrification. *Cryobiology* **52**, 228-240 (2006).
5. A. German, E. Y. Akdaş, Excitability and synaptic transmission after vitrification of mouse corticohippocampal slices. *bioRxiv* 10.1101/2024.06.03.597218, 2024.2006.2003.597218 (2024).
6. B. Wowk *et al.*, Vitrification Enhancement by Synthetic Ice Blocking Agents. *Cryobiology* **40**, 228-236 (2000).
7. B. Wowk, G. M. Fahy, Inhibition of bacterial ice nucleation by polyglycerol polymers. *Cryobiology* **44**, 14-23 (2002).
8. K. Hynynen, N. McDannold, N. A. Sheikov, F. A. Jolesz, N. Vykhodtseva, Local and reversible blood-brain barrier disruption by noninvasive focused ultrasound at frequencies suitable for trans-skull sonications. *Neuroimage* **24**, 12-20 (2005).
9. A. R. Rezai *et al.*, Focused ultrasound-mediated blood-brain barrier opening in Alzheimer's disease: long-term safety, imaging, and cognitive outcomes. *J Neurosurg* **139**, 275-283 (2023).
10. X. Lu *et al.*, Preserving extracellular space for high-quality optical and ultrastructural studies of whole mammalian brains. *Cell Rep Methods* **3**, 100520 (2023).
11. A. W. Sedar, J. G. Forte, EFFECTS OF CALCIUM DEPLETION ON THE JUNCTIONAL COMPLEX BETWEEN OXYNTIC CELLS OF GASTRIC GLANDS. *J Cell Biol* **22**, 173-188 (1964).
12. B. Cragg, Preservation of extracellular space during fixation of the brain for electron microscopy. *Tissue Cell* **12**, 63-72 (1980).
13. A. Gjedde, High- and low-affinity transport of D-glucose from blood to brain. *J Neurochem* **36**, 1463-1471 (1981).
14. N. MacAulay, Molecular mechanisms of brain water transport. *Nat Rev Neurosci* **22**, 326-344 (2021).
15. A. S. Verkman, A. J. Smith, P. W. Phuan, L. Tradtrantip, M. O. Anderson, The aquaporin-4 water channel as a potential drug target in neurological disorders. *Expert Opin Ther Targets* **21**, 1161-1170 (2017).
16. L. Tradtrantip, B. J. Jin, X. Yao, M. O. Anderson, A. S. Verkman, Aquaporin-Targeted Therapeutics: State-of-the-Field. *Adv Exp Med Biol* **969**, 239-250 (2017).
17. L. Unger *et al.*, AER-270 and TGN-020 are not aquaporin-4 water channel blockers. *bioRxiv* 10.1101/2024.12.04.625365, 2024.2012.2004.625365 (2024).
18. B. Yang, H. Zhang, A. S. Verkman, Lack of aquaporin-4 water transport inhibition by antiepileptics and arylsulfonamides. *Bioorg Med Chem* **16**, 7489-7493 (2008).
19. C. Sun *et al.*, Acutely Inhibiting AQP4 With TGN-020 Improves Functional Outcome by Attenuating Edema and Peri-Infarct Astrogliosis After Cerebral Ischemia. *Front Immunol* **13**, 870029 (2022).
20. Z. Han *et al.*, Vitrification and nanowarming enable long-term organ cryopreservation and life-sustaining kidney transplantation in a rat model. *Nat Commun* **14**, 3407 (2023).
21. E. Underwood, J. B. Redell, J. Zhao, A. N. Moore, P. K. Dash, A method for assessing tissue respiration in anatomically defined brain regions. *Scientific Reports* **10**, 13179 (2020).
22. P. Young *et al.*, Single-neuron labeling with inducible Cre-mediated knockout in transgenic mice. *Nat Neurosci* **11**, 721-728 (2008).
23. J. B. Singh *et al.*, Activity-dependent extracellular proteolytic cascade cleaves the ECM component brevican to promote structural plasticity. *EMBO reports* https://doi.org/10.1038/s44319-025-00644-w, 1-23-23 (2025).
24. P. Virtanen *et al.*, SciPy 1.0: fundamental algorithms for scientific computing in Python. *Nature Methods* **17**, 261-272 (2020).
